# Impact of the COVID-19 pandemic on delays in diagnosis and treatment of tick-borne diseases endemic to southeastern USA

**DOI:** 10.1186/s13071-023-05917-8

**Published:** 2023-08-24

**Authors:** Victor Arahirwa, Katherine Tyrlik, Haley Abernathy, Caitlin Cassidy, Aidin Alejo, Odai Mansour, Dana Giandomenico, Amanda Brown Marusiak, Ross M. Boyce

**Affiliations:** University of North Carolina at Chapel Hill, Chapel Hill, NC USA

**Keywords:** Spotted fever group rickettsiosis, Ehrlichiosis, Tick-borne disease, COVID-19

## Abstract

**Background:**

The Coronavirus disease 2019 (COVID-19) pandemic was marked by an increase in diagnosis and treatment delays for a range of medical conditions. Yet the impact of the pandemic on the management of tick-borne diseases, which frequently manifest as an acute febrile illness similar to COVID-19, has not been well described.

**Methods:**

In this retrospective cohort study of patients with suspected tick-borne disease attending the University of North Carolina Health facilities, we compared the timeliness of diagnosis and treatment in a “pre-COVID” period (March 2019 to February 2020) and a “post-COVID” period (March 2020 to February 2021). Participants included patients with an ICD-10 diagnosis code of spotted fever group rickettsiosis or ehrlichiosis and a positive *Rickettsia* *rickettsii* or *Ehrlichia* indirect immunofluorescence assay immunoglobulin G antibody test result. Of the 897 patients who had an eligible diagnosis, 240 (26.8%) met the inclusion criteria. The main outcome was time from initial presentation to definitive diagnosis and treatment.

**Results:**

During the 2-year study period, 126 (52.5%) patients were grouped in the pre-COVID period and 114 (47.5%) were grouped in the post-COVID period; 120 (50.0%) were female; and 139 (57.9%) were aged > 50 years. Comparing the post-COVID to the pre-COVID period, the adjusted odds ratio (aOR) for delay in treatment > 0 days was 1.81 (95% confidence interval [CI] 1.07–3.07, *P* = 0.03), and for a treatment delay > 7 days, 1.65 (95% CI 0.94–2.90, *P* = 0.08). The odds of a delay in diagnosis were similar for patients in the post- and pre-COVID periods, with an aOR of 1.61 (95% CI 0.96–2.72, *P* = 0.07) for delays > 0 days, and aOR of 1.72 (95% CI 0.99–3.00, *P* = 0.05) for delays > 7 days.

**Conclusions:**

The odds of a delay in treatment > 0 days were significantly higher in the post-COVID period than in the pre-COVID period. However, the odds of a delay in treatment > 7 days, or a delay in diagnosis, were similar between these two periods. Shifts in care-seeking, alternative care delivery models and prioritization of COVID-19 may contribute to diminished timeliness of treatment for patients with tick-borne diseases.

**Graphical Abstract:**

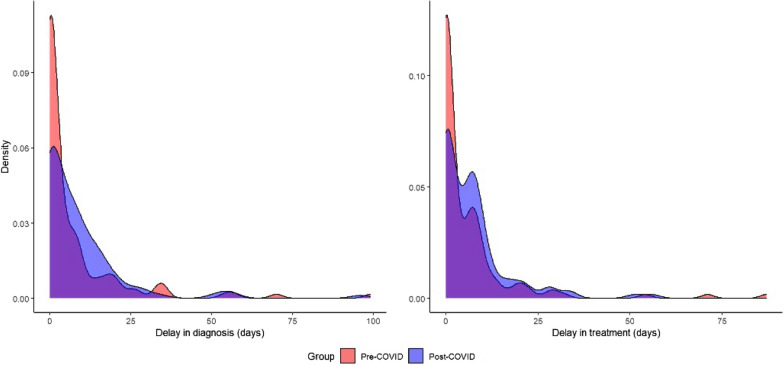

**Supplementary Information:**

The online version contains supplementary material available at 10.1186/s13071-023-05917-8.

## Background

From 2004 to 2016, nearly 500,000 cases of tick-borne disease (TBD) were reported to the US Centers for Disease Control and Prevention (CDC). Notably, the annual number of TBD cases doubled over the period of analysis with year-over-year increases in Lyme disease, ehrlichiosis and spotted fever group rickettsiosis (SFGR) [1]. In 2017 alone, a record number of TBD cases (*n* = 59,349) was reported to CDC, suggesting that these trends will continue [2]. However, the estimates, which are derived from notifiable disease reports, very likely underestimate the true incidence and burden of TBDs in the USA [3, 4].

While less frequently reported than Lyme disease, tick-borne rickettsiosis and ehrlichiosis are increasingly important public health threats [5], particularly in the Southeast region of the USA, where the Lone Star tick (*Amblyomma* *americanum*) is prevalent. This tick is a vector of *Ehrlichia* *chaffeensis* and *Ehrlichia* *ewingii*, both of which cause acute febrile illness that may progress to more severe and ultimately fatal disease within 7 to 14 days [6]. Rocky Mountain Spotted Fever (RMSF), caused by infection with *Rickettsia* *rickettsii,* is the most fatal tick-borne illness in the USA [7]. Similar nonspecific laboratory findings can be observed in both RMSF and ehrlichiosis, including transaminase elevation, thrombocytopenia and leukopenia [8]. Yet, the major factor driving morbidity and mortality associated with RMSF and ehrlichiosis remains delayed diagnosis and treatment [7, 9, 10].

The global emergence of the coronavirus disease 2019 (COVID-19) pandemic, with the first cases emerging in late 2019, caused substantial changes in care-seeking behaviors and access to health services. Notable changes affecting patients included deferral of acute and routine care, limited options to receive in-person evaluations and greater use of telemedicine, compounded by the requirement to test for severe acute respiratory syndrome coronavirus 2 (SARS-CoV-2) prior to hospitalization. A number of studies have demonstrated how these changes contributed to misdiagnosis and delayed care and ultimately led to worse patient outcomes across a range of conditions [11–15]. The underlying reasons for these delays are not well studied but are assumed to include patient fear of contracting the virus in healthcare settings, more limited evaluation attributable to telemedicine use and stay-at-home orders [16]. Furthermore, physicians might make diagnostic errors due to excessive concern about COVID-19, anchoring on the diagnosis at the expense of a broad differential for acute febrile illness syndromes [17].

Very little is known about the impact of the COVID-19 pandemic on the diagnosis and treatment of TBDs. In one small study, three patients with TBD experienced an increased duration of time between symptom presentation and diagnosis due to fears and restrictions related to the pandemic [18]. Similarly, a study in Poland showed a sizable decrease in the incidence of Lyme disease during the pandemic, which may suggest decreased care seeking and access to outdoor recreational spaces [19]. In one study of Lyme disease in the USA, participants spent more time outdoors and visited a CDC website describing safe tick removal more frequently in 2020 than in the preceding years. However, fewer persons sought care in the Emergency Department for tick bites and fewer laboratory tests for Lyme disease were ordered [20].

North Carolina (NC) experiences some of the highest incidence rates of SFGR and ehrlichiosis in the USA, often accounting for close to 16% and 9% of national totals reported to the CDC, respectively [5]. The state thus presents an interesting setting to examine potential impacts of the COVID-19 pandemic on the diagnosis and management of TBD [6]. Therefore, we sought to investigate pandemic-related health system changes that may have contributed to delays in the diagnosis and treatment of TBDs. Using a comparison of patients seen at two different time periods, this study addresses major limitations in previous studies, which have relied extensively on case reports with small sample sizes over shorter time frames.

## Methods

### Study design and participants

Patients with suspected SFGR or ehrlichiosis were identified utilizing the Carolina Data Warehouse for Health, a central repository containing clinical, research and administrative data sourced from University of North Carolina (UNC) Health, the largest academic health system in the state of North Carolina. In 2021, UNC Health reported 3.5 million clinical visits and 470,000 Emergency Department visits. Our study population included patients with an International Classification of Diseases, Tenth Revision (ICD-10) diagnosis code of SFGR (A77.0) or ehrlichiosis (A77.40) who were seen at any UNC Health facility utilizing Epic as the electronic medical record system in order to facilitate chart abstraction. Eligible periods included March 2019 to February 2020 (herein “pre-COVID”) and March 2020 to February 2021 (herein “post-COVID”). The post-COVID period approximates the first year of the pandemic as declared by the WHO on 11 March 2020 [21]. From this population, we selected the medical record numbers (MRN) of patients with a positive *R.* *rickettsii* or *Ehrlichia* indirect fluorescent immunoglobulin G (IgG) antibody test result (Biocell Diagnostics Inc., Baltimore, MD, USA), both of which exhibit substantial cross-reactivity across species (i.e. *R.* *rickettsii* and *R.* *parkeri*) [22]. PCR testing for *Ehrlichia* is available at UNC as a sendout to Mayo Clinic Laboratories but is not routinely ordered. Furthermore, PCR cannot be performed on stored serum (retrospective testing) [23].

Each MRN was reviewed to confirm that the corresponding individual met specific inclusion criteria (Table 1). These criteria were based on CDC case definitions for SFGR and ehrlichiosis [24, 25]. It should be noted that given the considerable exposure to *Rickettsia* *amblyommatis* and *E.* *ewingii*, and high seroprevalence of antibodies reactive with *R.* *rickettsii* and *E.* *chaffeensis* [22, 23], we used a higher immunofluorescence assay (IFA) IgG cutoff (≥ 1:256 compared to ≥ 1:128 for *R.* *rickettsii* and ≥ 1:64 for *E.* *chaffeensis*) and a more specific definition of probable cases than the CDC definition. For each eligible patient, we abstracted demographic and clinical information, including the results of laboratory testing. In addition, we documented: (i) the time in days from initial presentation to definitive diagnosis and treatment; (ii) the use of telehealth visits, if applicable; and (iii) the results of any severe acute respiratory syndrome coronavirus 2 (SARS-CoV-2) testing performed as part of the evaluation of symptoms. We also documented hospitalizations, intensive care unit (ICU) admissions, psychiatric referrals (included due to hypothesized increase in use of mental health services), sepsis and deaths during the course of a confirmed tick-borne illness.

**Table 1:** Study inclusion criteria

Criterion number	Description of criterion	Rationale
1	Presented to frontline provider during the period March 2019 to February 2020 or March 2020 to February 2021	One year prior to and 1 year after onset of pandemic
2	Age ≥ 18 years	Focus on adults only given presumed greater COVID-19 impact
3	Acute *Rickettsia* *rickettsii* or *Ehrlichia* serology performed within 14 days of symptom onset and convalescent serology performed within 14–70 days of acute serology, with fourfold change in titer between serologies	Individuals with confirmatory laboratory evidence
4	At least 2 clinical symptoms suspicious for TBD (e.g., fever, headache, rash or eschar, arthralgia or myalgia, nausea, vomiting, or diarrhea) and present for ≤ 14 days	Clinical presentation consistent with acute TBD infection
5	A single *R.* *rickettsii* or *Ehrlichia* test with a titer ≥ 1:256	Individuals with presumptive laboratory evidence
6	At least 1 *R.* *rickettsii* or *Ehrlichia* test without symptoms present for ≤ 14 days	Individuals with suspected TBD infection
7	No testing done and a leading diagnosis of TBD and a prescription of doxycycline	Individuals treated empirically

To be eligible for inclusion in the study, patients met criteria 1 and 2, plus either 3 and 4 (confirmed case); 4 and 5 (probable case); 6 (suspected case); or 7

*COVID-19* Coronavirus disease 2019, *TBD* tick-borne disease

### Statistical analysis

We calculated summary statistics for two primary outcomes, including the time from presentation to diagnosis and the time from presentation to treatment for the pre-COVID and post-COVID periods. We conducted Wilcoxon rank sum tests to compare the number of days until each outcome between the two periods. We carried out these tests twice, first defining a delay as > 0 days (i.e. not on initial encounter) and then again defining it as > 7 days, which has been shown to be associated with serious clinical outcomes [26]. Additionally, we conducted exact Mantel–Haenszel Chi-square tests (*χ*^2^) to test the hypothesis of no association between COVID period and sex, age group, health facility and insurance status. We also conducted Fisher’s exact tests to determine whether there was an association between pre- and post-COVID periods and each of five serious outcomes: hospitalization, sepsis, ICU admission, psychiatric referrals and death. All information was recorded in a case report (See Additional file 1: Figure S1) form using a secure electronic database.

We employed logistic regression to evaluate the relationship between the two periods and each of our primary outcomes. These outcomes were dichotomized in two ways: (i) if the time was > 0 days; and (ii) if the time was > 7 days. All models were adjusted for age group (e.g. < 50 vs ≥ 50 years) and sex as these variables were hypothesized to confound the association of interest. Odds ratios (OR) were calculated for delays, stratified by COVID period, age and sex, while adjusting for all other variables in the model. All analyses were conducted using SAS Studio 3.8 (SAS Studio Inc., SAS Institute, Cary, NC, USA). All figures were created using R 4.0.5 (The R Foundation for Statistical Computing, Vienna, Austria).

## Results

A total 897 patients had an eligible ICD diagnosis, of whom 240 (26.8%) met the inclusion criteria, including 126 (52.5%) in the pre-COVID period and 114 (47.5%) in the post-COVID period. Almost all of the excluded patients reported fewer than two symptoms. Of the 240 patients who met inclusion criteria, 14 (5.8%) had at least a fourfold increase in titer between acute and convalescent serologies; 124 (51.7%) had symptoms present for ≤ 14 days and only an acute sample with a titer ≥ 1:256; and 102 (42.5%) were treated empirically, even if symptoms were present for ≥ 14 days. The number of patients in each category was similar in the two periods.

The overall group was evenly divided between males and females (*N* = 120, 50.0%), but trended older with a majority of patients aged > 50 years(*N* = 139, 57.9%). The two groups were generally similar across sex, age, season of presentation and health insurance status. While outpatient visits were the most common care setting in both timeframes (52.4% pre-COVID and 57.5% post-COVID), more participants in the pre-COVID period (*N* = 41, 32.5%) were seen in an Emergency Department compared to the post-COVID period (*N* = 17, 15.0%). Telemedicine was only utilized by participants in the post-COVID timeframe, accounting for 11.4% (*N* = 13) of initial visits. No statistically significant associations were detected between pandemic group and sex, age category, health facility, duration of symptoms at first visit or insurance status. In the post-COVID period, 29 (25.4%) patients underwent one SARS-CoV-2 test while 11 (9.6%) underwent two tests; however, only three (7.0%) tests were positive for SARS-CoV-2 (See Additional file 1: Table S1).

**Fig. 1:**
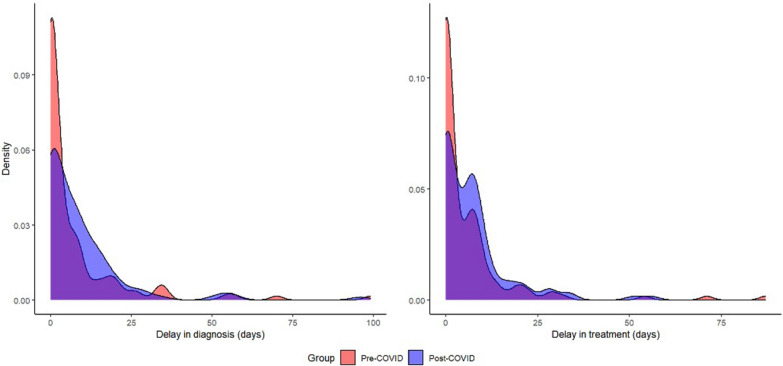
Distribution of delays in diagnosis and treatment for pre-COVID period (red) and post-COVID period (blue), with overlap indicated in purple. COVID, Coronavirus disease 2019

Differences in both time from presentation to diagnosis and time to treatment were detected when comparing periods. In the pre-COVID period, the median time from initial presentation to diagnosis was 1.0 (interquartile range [IQR] 0.0, 8.0) day, while in the post-COVID period the median time was 5.0 (IQR 0.0, 12.0) days (*P* = 0.03) (Table 2). Similarly, the median time from initial presentation to treatment in the pre-COVID period was 1.0 (IQR 0.0, 7.0) day, and in the post-COVID period it was 5.0 (IQR 0.0, 9.0) days (*P* = 0.01). When restricting the analyses to either delays > 0 days or delays > 7 days, however, the differences were not statistically significant. In the pre-COVID period, 37 out of 128 patients (28.9%) had delays in diagnosis > 7 days, and 42 out of 135 patients (31.1%) had delays in treatment > 7 days. In the post-COVID period, 47 out of 105 (44.8%) patients had delays in diagnosis > 7 days, while 49 out of 111 (44.1%) patients had delays in treatment > 7 days. More people in the pre-COVID period, however, received diagnosis and treatment on the day of initial presentation (i.e. delay of 0 days) than in the post-COVID period. Density plots reveal differences in time to both outcomes between the two periods (Fig. 1).

**Table 2:** Time to diagnosis and treatment

Time to diagnosis and treatment	Pre-COVID (*N* = 126)	Post-COVID (*N* = 114)	Overall (*N* = 240)
*Time from first presentation to diagnosis (days)*			
Mean (SD)	7.3 (14.8)	8.9 (13.9)	8.1 (14.4)
Median (IQR)	1.0 (0.0, 8.0)	5.0 (0.0, 12.0)	2.5 (0.0, 9.0)
Min–Max	0.0–99.0	0.0–96.0	0.0–99.0
Any delay (*P* value)			0.03*
Where delay > 0 days (*P* value)			0.37
Where delay > 7 days (*P* value)			0.034
*Time from first presentation to treatment (days)*			
Mean (SD)	5.9 (12.3)	7.3 (9.8)	6.6 (11.2)
Median (IQR)	1.0 (0.0, 7.0)	5.0 (0.0, 9.0)	2.5 (0.0, 8.0)
Min–Max	0.0–87.0	0.0–56.0	0.0–87.0
Any delay (*P* value)			0.01*
Where delay > 0 days (*P* value)			0.48
Where delay > 7 days (*P* value)			0.44

*IQR* Interquartile range,* Min-Max* minimum-maximum,* SD* standard deviation

^*^Statistically significant at *P*<0.05 according to Wilcoxon rank sum test

Logistic regression modeling showed an increase in the odds of a delay in treatment for the post-COVID period compared to the pre-COVID period (Table 3). Comparing the post-COVID to the pre-COVID period, the adjusted OR (aOR) for the delay in treatment > 0 days was 1.81 (95% confidence interval [CI] 1.07–3.07, *P* = 0.03) and 1.65 (95% CI 0.94–2.90, *P* = 0.08) for a treatment delay > 7 days. The odds of a delay in TBD diagnosis were similar when comparing the post- to pre-COVID periods, including aOR = 1.61 (95% CI 0.96–2.72, *P* = 0.07) for delays > 0 days and aOR = 1.72 (95% CI 0.99–3.00, *P* = 0.05) for delays > 7 days.

**Table 3:** Logistic regression results for delays in treatment and diagnosis

Delays in treatment and diagnosis	Adjusted OR (95% CI)	*P* value
*Outcome: delay in treatment > 0 days*		
Post-COVID vs Pre-COVID	1.81 (1.07, 3.07)	0.03*
Age 50+ vs < 50 years	0.99 (0.58, 1.68)	0.96
Female vs Male	1.21 (0.72, 2.05)	0.47
*Outcome: delay in treatment > 7 days*		
Post-COVID vs Pre-COVID	1.65 (0.94, 2.90)	0.08
Age 50+ vs < 50 years	1.17 (0.66, 2.08)	0.59
Female vs Male	1.80 (1.02, 3.19)	0.04
*Outcome: delay in diagnosis > 0 days*		
Post-COVID vs Pre-COVID	1.61 (0.96, 2.72)	0.07
Age 50+ vs < 50 years	1.54 (0.91, 2.60)	0.11
Female vs Male	0.84 (0.50, 1.41)	0.51
*Outcome: delay in diagnosis > 7 days*		
Post-COVID vs Pre-COVID	1.72 (0.99, 3.00)	0.05
Age 50+ vs < 50 years	1.32 (0.75, 2.31)	0.34
Female vs Male	0.86 (0.50, 1.50)	0.61

All models were adjusted for age and sex

*CI* Confidence interval, *OR* odds ratio

*Statistically significant at* P* < 0.05

In the pre-COVID period there were 19 (15.1%) hospitalizations and two (1.6%) deaths associated with TBD compared to 16 (14.0%) hospitalizations and zero deaths in the post-COVID period (Table 4). Using Fisher’s exact test to compare the five severe outcomes of hospitalization, sepsis, ICU admission, psychiatric referrals, and death, we did not find any significantly different rates of serious clinical outcomes between the two periods.

**Table 4:** Serious outcomes by period of analysis and Fisher’s exact test results

Serious outcomes	Pre-COVID (*N* = 126)	Post-COVID (*N* = 114)	Overall (*N* = 240)	Fisher’s exact *P* value
Hospitalizations,* n* (%)	19 (15.1)	16 (14.0)	35 (14.6)	0.86
Sepsis,* n* (%)	3 (2.4)	2 (1.8)	5 (2.1)	*P* > 0.99
ICU admissions,* n* (%)	5 (4.0)	6 (5.3)	11(4.6)	0.72
Psychiatric referrals,* n* (%)	3 (2.4)	5 (4.4)	8 (3.3)	0.48
Deaths,* n* (%)	2 (1.6)	0 (0.0)	2 (0.8)	0.50

*ICU* Intensive care unit

## Discussion

Our study identified treatment delays during the COVID-19 pandemic that may have affected the delivery of care to patients with TBDs. Specifically, we observed decreased utilization of Emergency Department care and increased uptake of telemedicine services. In addition, patients seeking care in the post-COVID period often had to undergo testing for SARS-CoV-2 before being seen in-person or evaluated for alternative etiologies of acute febrile illness. Overall, these changes likely contributed to the significant delays in treatment observed in the post-COVID period.

There are a number of factors that may further explain these findings. Foremost among these is the non-specific nature and overlap of COVID-19 and TBD symptoms (e.g. fever, headache, fatigue). Given the heightened awareness of COVID-19 during the study period, providers may have anchored on a COVID-19 diagnosis on initial presentation, delaying the consideration of TBD and empirical treatment while SARS-CoV-2 testing was performed. We recommend that providers broaden their differential in a patient with acute febrile illness, and to suspect SFGR or ehrlichiosis in the setting of relevant epidemiologic exposure, tick season, typical clinical manifestations and laboratory test abnormalities [27, 28].

Furthermore, decreased access to outpatient clinics, hesitation about going to the Emergency Department due to long waits and fears of contracting SARS-CoV-2 and increasing utilization of telemedicine appointments may have negatively impacted the quality of care [18]. Telemedicine visits may have affected the history taking and all but negated the physical examination, which may have contributed to missing key elements of the illness. For example, providers may have failed to identify an eschar or rash during telemedicine visits.

Given the risk of rapid progression, it was reassuring that a large proportion of patients received a diagnosis and/or treatment at initial presentation [7]. Only 14 (5.8%) patients presented with clinical criteria for acute infection and serologic evidence of a fourfold increase in IgG-specific antibody titer between paired serum specimens. In other words, most patients were diagnosed and treated without the complete results of laboratory testing. This pattern is consistent with that observed in other studies [29], and reflects the importance of empirical treatment for patients with symptoms and exposure history concerning for RMSF. Conversely, a few patients had delays in diagnosis (3.4%) and/or treatment (2.0%) > 50 days. We suspect this could be due to issues such as documentation errors or loss to follow-up.

The fact that we did not observe any differences in important outcomes, including hospitalization, ICU admission or death, partly reflects the relative infrequency of these events. Some of this infrequency may be attributable to infection with less pathogenic rickettsial pathogens such as *R.* *parkeri*, which has serologic cross-reactivity with *R.* *rickettsii* [22]. Additionally, the delays in diagnosis and treatment that we observed were relatively modest and likely were not sufficient to cause systemic disease manifesting as vasculitis and end-organ damage that are associated with mortality. However, our findings cannot exclude the possibility that delays adversely affected some of the individuals in our study.

To our knowledge, this study is the largest and most robust analysis of potential impacts of the COVID-19 pandemic on the management of TBD. Our study has a number of strengths, including the use of a high-quality database, rigorous eligibility criteria, access to the complete medical record and location in North Carolina, where rates of SFGR and ehrlichiosis are among the highest in the nation. The study also has several limitations. First, while ours is the largest study to date, the sample size was still relatively small, especially in regard to our analysis of serious outcomes. Second, the study population was drawn from patients seen at a large academic medical system. Therefore, patient care experience may not reflect that of other health facilities, such as community hospitals or private practices that were more severely impacted by the pandemic. Lastly, our study cohort skewed towards older patients, who may have been more likely to develop overt clinical symptoms or have more health concerns related to age, given that this was an established and well-published risk factor for severe COVID-19. However, we note that the cohort looks demographically similar to that of our previous studies conducted prior to the pandemic [29].

Future research directions include comparing pre-pandemic data to data from later in the pandemic (e.g. 2022–2023) to assess for a reversion to pre-pandemic care patterns, assessing the effect of the pandemic on TBDs endemic to other geographical regions, such as Lyme disease, investigating delays in diagnosis and treatment of TBDs in non-academic settings and using PCR-based testing to diagnose SFGR and ehrlichiosis.

## Conclusions

Our study found delays in the diagnosis and treatment of SFGR and ehrlichiosis during the COVID-19 pandemic compared to the pre-COVID period. Key factors driving these delays include a shift in care-seeking, care delivery models and prioritization of COVID-19 as a potential cause of illness. These concerning findings highlight the effect of COVID-19 on the diagnosis and treatment of TBDs and merit both further study and educational interventions to minimize preventable morbidity and mortality attributable to TBD.

### Supplementary Information

**Additional file 1: Table S1**. Results of serological testing for SARS-CoV-2, *R.* *rickettsii* and *Ehrlichia*. **Figure S1.** Case report form for health system activities and patient outcomes.

## Data Availability

All data supporting the findings of this study are available within the paper and its supplementary information.

## Abbreviations

AOR: Adjusted odds ratio
CDC: US Centers for Disease Control and Prevention
COVID-19: Coronavirus disease 2019
ICD-10: International Classification of Diseases, Tenth Revision
IFA: Immunofluorescence assay
IgG: Immunoglobulin G
ICU: Intensive care unit
OR: Odds ratio
RMSF: Rocky Mountain spotted fever
SARS-CoV-2: Severe acute respiratory syndrome coronavirus 2
SFGR: Spotted fever group rickettsiosis
TBD: Tick-borne disease
UNC: University of North Carolina
