# Supplementary material for: Impact of the COVID-19 pandemic on delays in diagnosis and treatment of tick-borne diseases endemic to southeastern USA
Source: Parasit Vectors. 2023 Aug 24;16:295. doi: 10.1186/s13071-023-05917-8 (PMC10463840; doi:10.1186/s13071-023-05917-8)
Supplement: Supplementary file 1 — Additional file 1: Table S1. Results of serological testing for SARS-CoV-2, R. rickettsii and Ehrlichia. Figure S1. Case report form for health system activities and patient outcomes. [file 13071_2023_5917_MOESM1_ESM.docx]

**Table S1. Results of serological testing for SARS-CoV-2, *R. rickettsii*, and *Ehrlichia***

|  | **Pre-COVID**  **N=126**  **N (%)** | **Post-COVID**  **N=114**  **N (%)** | **Overall**  **N=240**  **N (%)** |
| --- | --- | --- | --- |
| **SARS-CoV-2 Test** | 0 | 43 (37.7) | 43 (17.9) |
| Positive | 0 | 3 (7.0) | 3 (1.3) |
| **Acute Test for *R. rickettsii*** | 103 (81.7) | 101 (88.6) | 204 (85.0) |
| Positive | 92 (89.3) | 89 (88.1) | 181 (88.7) |
| 1:64 | 10 (11.0) | 13 (14.6) | 23 (12.7) |
| 1:128 | 34 (37.4) | 28 (31.5) | 62 (34.3) |
| 1:256 | 33 (36.3) | 33 (37.1) | 66 (36.5) |
| 1:512 | 6 (6.6) | 9 (10.1) | 15 (8.3) |
| ≥1:1024 | 8 (8.8) | 6 (6.7) | 14 (7.8) |
| **Convalescent Test for *R. rickettsii*** | 59 (57.3) | 62 (61.4) | 121 (59.3) |
| Positive | 54 (91.5) | 56 (90.3) | 110 (90.9) |
| 1:64 | 12 (22.2) | 13 (23.2) | 25 (22.7) |
| 1:128 | 16 (29.6) | 14 (25.0) | 30 (27.3) |
| 1:256 | 26 (29.6) | 19 (33.9) | 35 (31.8) |
| 1:512 | 4 (7.4) | 5 (8.9) | 9 (8.2) |
| ≥1:1024 | 6 (11.1) | 5 (8.9) | 11 (10.0) |
| **Acute Test for *Ehrlichia*** | 79 (62.7) | 94 (82.5) | 173 (72.1) |
| Positive | 48 (60.8) | 66 (70.2) | 114 (65.9) |
| 1:64 | 9 (18.8) | 18 (27.3) | 27 (23.7) |
| 1:128 | 16 (33.3) | 19 (28.8) | 35 (30.7) |
| 1:256 | 12 (25.0) | 17 (25.8) | 29 (25.4) |
| 1:512 | 6 (12.5) | 8 (12.1) | 14 (12.3) |
| ≥1:1024 | 5 (10.4) | 4 (6.1) | 9 (7.9) |
| **Convalescent Test for *Ehrlichia*** | 31 (36.9) | 30 (31.9) | 61 (35.3) |
| Positive | 26 (83.9) | 24 (80.0) | 50 (82.0) |
| 1:64 | 7 (26.9) | 7 (29.2) | 14 (28.0) |
| 1:128 | 9 (34.6) | 10 (41.7) | 19 (38.0) |
| 1:256 | 4 (15.4) | 3 (12.5) | 7 (14.0) |
| 1:512 | 1 (3.8) | 3 (12.5) | 4 (8.0) |

**Fig. S1. Case report form for health system activities and patient outcomes**
